# Toll-like Receptor Signaling Activation by *Entamoeba histolytica* Induces Beta Defensin 2 in Human Colonic Epithelial Cells: Its Possible Role as an Element of the Innate Immune Response

**DOI:** 10.1371/journal.pntd.0002083

**Published:** 2013-02-28

**Authors:** Jorge-Tonatiuh Ayala-Sumuano, Victor M. Téllez-López, M. del Carmen Domínguez-Robles, Mineko Shibayama-Salas, Isaura Meza

**Affiliations:** 1 Department of Molecular Biomedicine, Centro de Investigación y de Estudios Avanzados del Instituto Politécnico Nacional, México DF, México; 2 Department of Infectomics and Molecular Pathogenesis, Centro de Investigación y de Estudios Avanzados del Instituto Politécnico Nacional, México DF, México; Georgetown University, United States of America

## Abstract

**Background:**

*Entamoeba histolytica*, a protozoan parasite of humans, produces dysenteric diarrhea, intestinal mucosa damage and extraintestinal infection. It has been proposed that the intestinal microbiota composition could be an important regulatory factor of amebic virulence and tissue invasion, particularly if pathogenic bacteria are present. Recent *in vitro* studies have shown that *Entamoeba histolytica* trophozoites induced human colonic CaCo2 cells to synthesize TLR-2 and TLR-4 and proinflammatory cytokines after binding to the amebic Gal/GalNac lectin carbohydrate recognition domain. The magnitude of the inflammatory response induced by trophozoites and the subsequent cell damage were synergized when cells had previously been exposed to pathogenic bacteria.

**Methodology/Principal Findings:**

We show here that *E. histolytica* activation of the classic TLR pathway in CaCo2 cells is required to induce β defensin-2 (HBD2) mRNA expression and production of a 5-kDa cationic peptide with similar properties to the antimicrobial HBD2 expressed by CaCo2 cells exposed to enterotoxigenic *Escherichia coli*. The induced peptide showed capacity to permeabilize membranes of bacteria and live trophozoites. This activity was abrogated by inhibition of TLR2/4-NFκB pathway or by neutralization with an anti-HBD2 antibody.

**Conclusions/Significance:**

*Entamoeba histolytica* trophozoites bind to human intestinal cells and induce expression of HBD2; an antimicrobial molecule with capacity to destroy pathogenic bacteria and trophozoites. HDB2's possible role as a modulator of the course of intestinal infections, particularly in mixed ameba/bacteria infections, is discussed.

## Author Summary

*Entamoeba histolytica* ameba/bacteria mixed intestinal infections are common in endemic regions of Amebiasis. Recent investigations support the idea that pathogen interplay in these infections may have a role in invasive disease, activating signals that increase intestinal inflammation. We have studied interactions of amebic trophozoites with human colonic CaCo2 cells, using as positive control pathogenic intestinal bacteria *E. coli* (ETEC). Both pathogens activated a chain of chemical reactions in the cells that led to production of the antimicrobial peptide β defensin-2 (HBD2), an element of the innate immune response. Pathogen activation of CaCo2 cell response and production of HBD2 were analyzed employing biochemical, cell, molecular biology, and immunology methods. Amebas induced HBD2 via the same classic Toll-receptor signaling pathway activated by ETEC. Amebic-induced HBD2 showed capacity to permeabilize and cause severe damage to bacteria and ameba membranes. Although this study was done *in vitro*, due to lack of an adequate animal model in which to monitor ameba/bacteria interactions, it provides a new insight into intestinal infections, showing that presence of amebas induces synthesis of elements of an innate immune response that could affect the equilibrium of the intestinal microbiota and modify the course of intestinal infections by other pathogens.

## Introduction

Diarrheic diseases annually cause 1.87 million deaths of children below 5 year old, of which 73% occur in 15 developing countries, including Mexico [1]. International reports have estimated that 100,000 deaths/year attributed to these diseases are caused by complications due to invasive amebiasis [2], [3]. Mixed infections of *Entamoeba histolytica* and pathogenic bacteria have been reported in endemic regions of amebiasis [4], [5], [6]. In 32% of the cases of acute diarrhea in Bangladesh children, the most frequently identified pathogens, besides *E. histolytica* and *E. dispar* trophozoites, were enterotoxigenic *Escherichia coli* (ETEC), *Salmonella sp* and *Shigella sp* [7]. Recent experimental data have provided support to the idea that presence of enteropathogenic bacteria and *E. histolytica* in mixed infections may play an important role in the establishment of invasive disease, by increasing adhesion, chemotaxis and cell damage capacity of trophozoites [8], [9], [10].

It is well known that interactions between microorganism of the intestinal flora and diverse molecules in the intestinal epithelium surface are precisely regulated in order to maintain intestinal homeostasis [10], [11], [12]. Binding of microbial surface molecules, known as specific pathogen-associated molecular patterns or PAMPS, to epithelial cell Toll-like receptors (TLRs) triggers activation of several signaling pathways relevant to intestinal inflammation [13]. One of these pathways activates transcription factors such as NFκB, AP1 and IRF that, in turn, can induce expression of proinflammatory cytokines such as IL-8, IL-1β, TNF-α and IFN. In addition, antimicrobial peptides such as cathelicidins and defensins are produced as response of the organism against the presence of intestinal pathogens [14], [15].

Human colonic epithelial CaCo2 cells form confluent monolayers that maintain *in vitro* epithelial barrier functions regulated by intercellular membrane junctions to preserve their polarization and selectivity in the transport of ions and other molecules [16], [17]. CaCo2 cells also express on their surface several receptors of PAMPs, including TLR-2 and TLR-4 [9]. It has also been shown, that binding of *E. histolytica* trophozoites to CaCo2 cells activates the classic pathway of TLR signaling, in which the activated form of NFκB induces transcription of proinflammatory cytokines and TLR-2 and TLR-4 genes for the production of the corresponding proteins. Although no data are available about trophozoite-mediated induction of intestinal antimicrobial defensins, as it would be in an innate immune response, the above findings make plausible to think that this induction may occur in CaCo2 cells.

During an intestinal inflammation caused by microbes several antimicrobial molecules are produced, being β defensins 1, 2 and 3 the most common in these cells [18], [19]. Defensins are cysteine-rich cationic peptides of low molecular weight, 3 to 5 kDa, that bind to microbe biological membranes rich in anionic phospholipids [20], [21]. Defensins integrate into the membranes inducing strain within the lipid bilayer, followed by a phase transition and the formation of pores [22]. It is also reported that in target prokaryotic cells and some protozoan parasites, defensins induce disruption of membrane Na+/K+ channel activity and phosphatidylserine exposure [23], [24]. Increased expression of HBD2 and HBD3 by intestinal cells in the presence of Gram-negative pathogenic bacteria has also been correlated with increased antibacterial activity [25], [26].

In the present work, we demonstrate that *E. histolytica* trophozoites induce, in CaCo2 cells and through the TLR2/4-NFκB pathway, the expression and release of an antimicrobial peptide with similar properties to HBD2 induced by ETEC in these cells and utilized here as reference. Functional characterization of the peptide showed its capacity to permeabilize HBD2-sensitive pathogenic bacteria as well as live trophozoites, which membranes showed evident structural alterations. The importance of HBD2 induction by trophozoites and bacteria in intestinal infections is discussed in the context of recent evidence which has shown that, an inflammatory milieu and the immune response elicited to control pathogens also disrupts the intestinal epithelial barrier, allowing penetration of more pathogens, produces dysbiosis and the creation of a favorable environment for pathogen invasion [27].

## Methods

### Cell culture

Human colonic epithelial CaCo2 cells (ATCC HTB-37) were cultured in DMEM containing 10% fetal bovine serum, 1% streptomycin and 1% penicillin (Gibco, Grand Island, NY, USA) at 37°C and 5% CO_2_. *Entamoeba histolytica* trophozoites (strain HM1-IMSS) were cultured in TY1-S-33 medium [28], after passage in hamster liver and tested for induction of well-defined tissue lesions. Bacteria utilized in this work corresponded to: a clinical isolate of enterotoxigenic *Escherichia coli* (ETEC H10407 078: H11), containing genes codifying for thermolabile toxin (LT), thermostable toxin (ST) and colonization factor (CFA), kindly donated by Dr. Teresa Estrada (CINVESTAV-IPN, Mexico) and *Staphylococcus aureus* obtained from the CINVESTAV-IPN Certified Bacterial Strain Reservoir. All bacteria were cultured in LB broth.

### Ethics statement

Animal used in this study were handled according to the protocol 423-08 approved by the Institutional Committee (IACUC) of Centro de Investigación y de Estudios Avanzados del IPN (CINVESTAV). Our institution fulfills the technical specifications for production, care and use of laboratory animals and is certified by federal law (NOM-062-ZOO-1999). Hamsters were killed by an overdose of sodium pentobarbital and handled according to guidelines of the 2000 AVMA Panel of Euthanasia.

### Exposure of CaCo2 cells to pathogens

Confluent cultures of CaCo2 cells were switched to DMEM containing only 1% fetal bovine serum for 12 h. Then, cells were exposed for 2 h to *Entamoeba histolytica* trophozoites (in a 1∶2 cell/ameba ratio) that were previously treated for 20 min with 3.7% electron microscope grade paraformaldehyde (PFA) in PBS at room temperature, to avoid cell killing by amebas, and thoroughly rinsed with ice-cold PBS, or were exposed to ETEC in a 1∶100 cell/bacteria ratio for 2 h at 37°C and then treated to remove the bacteria [9]. The number of trophozoites employed was determined in Neubauer chambers and the number of bacteria (from overnight cultures) by spectrophotometry at 600 nm (Smart Spec 3000, Bio-Rad Laboratories Inc., Hercules, CA, USA). The inhibitor of NFκB activation, Bay117085, was added to the culture medium 30 min prior to exposure to pathogens at a final concentration of 20 µM. IMG-2005-5, inhibitor of MyD88, was added at a final concentration of 100 µM for 24 h prior exposure to pathogens. After incubation for 2 h in the above conditions, conditioned media or CM (a term commonly used to designate culture media in which cells have been cultured for an established time and into which components from the cells have been released and metabolized) were recovered, centrifuged at 12,000×rpm and filtered through 0.22 µm Millipore filters, kept at −70°C after protein determination and used within 2 weeks. Endotoxin values, quantified utilizing the *Limulus* Amebocyte kit (Lonza Group Ltd, Basel, Switzerland), for CaCo2 cell culture medium (DMEM) and CM obtained from CaCo2 cells exposed to PFA-fixed trophozoites were in the range of 0.01–0.02 EU/µg. The chromatography-enriched fraction of HBD2 (CECE-HBD2) showed values<0.01 EU/µg. HBD2 concentration in CM of cells exposed to pathogens was determined by ELISA using a specific rabbit polyclonal anti-HBD2 antibody (Santa Cruz Biotechnology, Santa Cruz, CA, USA) and by densitometry of the protein band identified after SDS-PAGE and immunoblotting.

### Cation exchange chromatography

Caco2 cells were seeded in 75 cm^2^ culture bottles and exposed to ETEC as indicated. The supernatants from 20 bottles (150 ml) were collected, centrifuged and filtered through 0.22 µm Millipore filters and after addition of a cocktail of protease inhibitors (Complete, Roche Applied Science, Mannheim, Germany), lyophilized and stored at −70°C. Supernatants from control cells, not exposed to bacteria, were obtained in the same way. Chromatography was performed in a cation exchange column of methacrylate copolymer containing sulfuric anhydride functional groups (Macro-prep High S, Bio-Rad). The resin was separated from the ethanol (used as preserver) by decantation and washed with deionized water. To prepare the column, the resin was suspended in 60 volumes of 0.0172 M potassium acetate adjusted to pH, 5.3 with acetic acid (Solution 1). Lyophilized samples were reconstituted in 15 ml of Solution 1, mixed with the resin and gently stirred for 16 h at 4°C. The resin was washed with Solution 1 by decantation and the column mounted. Elution of bound peptides was done with 6 volumes of solution 1 containing 1.0 M NaCl, pH 5.3. Fractions of 1 ml were collected and protein-containing fractions (determined by 280 nm spectrometry) pooled together. Desalinization of the pooled fractions was done employing the Clean up Kit (Bio-Rad). Cation exchange chromatography-enriched HBD2 (CECE-HBD2) was reconstituted in PBS, aliquoted and kept at −70°C.

### Electrophoresis and immunoblotting

Conditioned media (CM) obtained from pathogen-exposed and non-exposed CaCo2 cells (referred as control cell in the assays performed) as well as synthetic HBD2 peptide obtained from Princeton Biomolecule (Langhorne, PA, USA) were separated in 15% gels by SDS-PAGE and stained with silver nitrate. Parallel gels were processed for immunoblotting. Blotted nitrocellulose membranes were challenged with a rabbit polyclonal anti-HBD2 antibody (Santa Cruz Biotechnology, Santa Cruz, CA, USA) at a 1∶500 dilution for 12 h at 4°C followed by horseradish peroxidase-conjugated sheep anti-rabbit IgG antibody (Invitrogen, Carlsbad, CA, USA) at 1∶8000 dilution for 2 h at room temperature.

### Quantitative RT-PCR

Total RNA was extracted from CaCo2 cells that were exposed or non-exposed to pathogens using Trizol (Invitrogen). The reverse transcriptase reaction was carried out from 1 µg of RNA utilizing the Superscript II synthesis system (Invitrogen) following the specifications of the manufacturer. For quantitative PCR assays the SYBR Green PCR Master Mix Kit (Applied Biosystems, Warrington, UK) was used. PCR was run in a Thermalcycler 7000 (Applied Biosystems). For determination of HBD2 expression (gene: *defb4a*; Acc. No. NM_004942) the specific primers utilized were: sense 5′-CATCAGCCATGAGGGTCTTGT-3′ and anti-sense 5′-GAGACCACAGGTGCCAATTT-3′ with an annealing temperature of 55°C. As normalizing factor, expression of the Ribosomal Protein Large P0 (gene: *rplp0;* Acc. No. NM_001002) was evaluated by using the specific primers sense 5′-CAGATTGGCTACCCAACTGTT-3′ and antisense 5′-GGGAAGGTGTAATCCGTCTCC-3′ with an annealing temperature of 61°C. Expression levels of HBD2 mRNA were determined using 2^−ΔΔCt^ formula. Both primer pairs were evaluated for their efficiency in serial dilution PCR reactions. Efficiency fitted between 1.95 and 2.05. Additional data about the primers used is presented in figure S1.

### Flow cytometry

Flow cytometry analysis was employed to assess HBD2 retained inside CaCo2 cells exposed to the pathogens. Assays were performed in the presence of 1.0 µg/ml of Brefeldin A (BFA) to inhibit release of HBD2 into the culture medium. Pathogen exposed cells were extensively rinsed and given a 20 min treatment with 5 mM sodium azide and 50 µg/ml of gentamycin followed by extensive rinsing with PBS to remove the pathogens. CaCo2 cells were then detached from the culture dishes using trypsin, centrifuged and FACS permeabilizing solution (Becton Dickinson, Mountain View, CA, USA) added to the pellet for 10 min at RT to fix and permeabilize the cells. After washing by centrifugation with PBS containing 2% FBS, cells were resuspended and incubated with anti-HBD2 antibody (1∶500 dilution) for 12 h at 4°C and then with a secondary antibody coupled to FITC (1∶8000 dilution) for 2 h at room temperature. Cells were washed with PBS and analyzed in a FACScalibur flow cytometer (Becton Dickinson) to determine fluorescence intensity.

### Viability assays

One hundred thousand live trophozoites were resuspended in 1 ml of PBS containing a final concentration of 10 ng of CECE-HBD2 and gently mixed for 2 h at 37°C. In parallel, the same number of live trophozoites was resuspended in CM from CaCo2 cells previously exposed to *Entamoeba histolytica* trophozoites or to ETEC, or resuspended in CM from control cells not exposed to pathogens. Trophozoites were incubated at 37°C for 2 h with gentle mixing. After rinsing the trophozoites with PBS at 37°C, these were resuspended in 500 µl of PBS and propidium iodide added to a final concentration of 1.0 µg/ml and immediately analyzed for fluorescence intensity by flow cytometry or prepared for electron microscopy. Neutralization of HBD2 present in CM was determined after addition of the anti-HBD2 antibody (2.0 µg/ml) to the trophozoite incubation mixture and propidium iodide, as indicated above, before trophozoites were analyzed by flow cytometry. As control, 2.0 µg/ml of an unrelated polyclonal antibody (MBL International. Woburn, MA) directed to human Sirt-1 protein were added to a parallel incubation mixture. *Staphylococcus aureus* cells (100,000 for each assay condition) were treated the same way as indicated for trophozoites and used as positive control for HBD2 antimicrobial activity [24], [26].

### Transmission electron microscopy

For ultrastructural analysis, trophozoites that had been exposed to CM or to CECE-HBD2 were washed with PBS and twice with 0.1 M sodium cacodylate buffer and fixed for 2 h with 2.5% glutaraldehyde in 0.1 M sodium cacodylate buffer, pH 7.4. Fixed trophozoites were then washed twice with 0.1 M sodium cacodylate buffer, post-fixed with 2.0% osmium tetroxide, dehydrated with ethanol at increasing concentrations, and treated with propylene oxide. The trophozoites were then embedded in epoxy resins. Semi-thin sections were stained with toluidine blue for light microscopic examination. Thin sections were stained with uranyl acetate followed by lead citrate and examined with a Zeiss EM-10 transmission electron microscope.

### Immunofluorescence of NFκB

To assess the translocation and activation of the transcription factor NFκB, CaCo2 cells were exposed to trophozoites or ETEC bacteria in the absence or presence of inhibitors of the classic TLR pathway. After pathogen exposure, cells were fixed with PFA and permeabilized. The translocation of NFκB was evidenced by utilizing a specific anti-NFκB p65 subunit antibody (Santa Cruz Biotechnology) and a FITC-tagged secondary antibody. Cell nuclei were revealed by DAPI staining.

### Statistical analysis

Data are presented as mean ± standard deviation. Mann-Whitney test for the comparison of two data groups and Kruskal-Wallis test were chosen to analyze statistical differences. Statistical differences were set with a *P* value equal or lower than 0.05 as determined by the tests. Each result represents at least two independent experiments done in triplicate.

## Results

### Exposure of human colonic CaCo2 cells to *Entamoeba histolytica* trophozoites induces expression of HBD2 mRNA

Previous studies with intestinal epithelial cells had shown that secretion of antimicrobial peptides, particularly defensins, is induced after activation of TLR2/4 and IL-1R signaling pathways by enteropathogenic bacteria [19], [29], [30]. Recent studies with CaCo2 cells [9] had shown that classic TLR signaling is activated after binding of *E. histolytica* trophozoites to TLR2 and TLR4 receptors in these cells. Binding occurs through the carbohydrate recognition domain of the amebic Gal/GalNac lectin. This molecule is an important molecular component of the parasite surface and also a stimulator of cytokine production by CaCo2 cells and macrophages [9], [31], [32]. Considering the above results, we hypothesized that expression of the antimicrobial HBD2 could be induced in human colonic cells by interaction with *E. histolytica* trophozoites.

**Figure 1:**
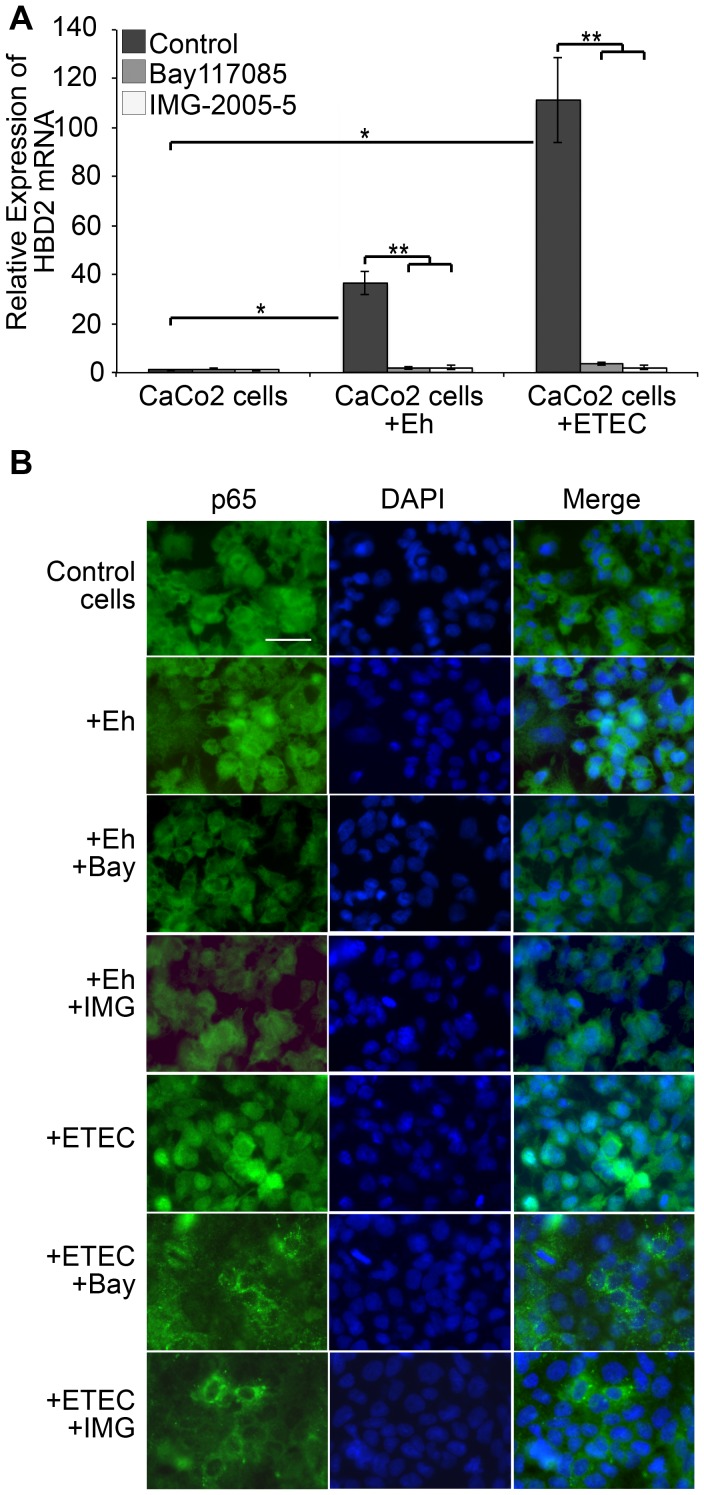
HBD2 mRNA expression is induced by *Entamoeba histolytica* trophozoites in CaCo2 cells by activation of TLR2/4 classic pathway. A. CaCo2 cells were exposed to PFA-fixed *E. histolytica* trophozoites (Eh) in a 1∶2 ratio for 2 h in culture media containing only 1% serum. Cells exposed to Enterotoxigenic *Escherichia coli* (ETEC) in a 1∶100 ratio, in the same conditions, were used as reference or positive control of HBD2 mRNA induction. After pathogen exposure, CaCo2 cells were washed extensively as indicated in Methods before been lysed for total RNA isolation. Expression of HBD2 mRNA was measured by relative quantitative RT-PCR. To investigate the participation of the classic pathway of TLR2/4 in the induction of HBD2 mRNA expression, CaCo2 cells exposed to pathogens were incubated with the inhibitors of NFκB activity, Bay117085, or MyD88 signaling, IMG-2005-5, as described in Methods section. Data are presented as fold change relative to control CaCo2 cells ± SD from two independent experiments done in triplicate. * Indicates statistical differences in CaCo2 cells exposed to pathogens in comparison to non-exposed cells, *P* value<0.01. ** Indicates statistical differences between expression of HBD2 mRNA in cells exposed to the same pathogen in presence or absence of the inhibitors. *P* value<0.001. B. Translocation of NFκB p65 subunit to nuclei of CaCo2 cells after exposure to *Entamoeba histolytica* trophozoites. Cells treated as indicated in A were fixed and permeabilized for detection of NFκB p65 subunit with a specific antibody and a secondary antibody tagged with FITC. Nuclei were visualized by DAPI staining. Cells exposed to *E. histolytica* (+Eh), cells exposed to ETEC (+ETEC), cells exposed to pathogens in presence of inhibitors BAY117085 (+Bay) or IMG-2005-5 (+IMG). Bar = 20 µm.

To test this hypothesis, CaCo2 cells were exposed to PFA-fixed trophozoites in a 1∶2 ratio for 2 h or to ETEC bacteria in a 1∶100 ratio for the same length of time. As induction of HBD2 in CaCo2 cells has already been reported to occur when they are exposed to ETEC [30], cells exposed to these bacteria were used as positive control for HBD2 induction by amebas. Total RNA was isolated from cells either not exposed to pathogens (control cells) or from cells exposed to pathogens and HBD2 mRNA expression evaluated by real time quantitative RT-PCR. Expression of constitutive *rplp0* mRNA was evaluated as normalizing factor. Primers used and controls for the quantitative RT-PCR assays are shown in supplementary material (Supplemental Figure S1). Figure 1A shows that when CaCo2 cells were exposed to ETEC (positive control), the relative expression of HBD2 mRNA increased close to 100-fold, compared to its baseline expression in cells not exposed to pathogens. Exposure of CaCo2 cells to PFA-fixed *E. histolytica* trophozoites increased expression of HBD2 mRNA close to 38-fold, compared to the baseline expression of control cells. When the expression of HBD2 mRNA was evaluated in presence of inhibitors that block the TLR-NFκB pathway, a strong negative effect was evident in the expression of the defensin mRNA.

Additional evidence to support activation of TLR signaling in this process was obtained monitoring the activation of NFκB p65 subunit translocated to the cell nuclei. Figure 1B shows localization of the anti-p65 antibody signal (green) and DAPI-stained nuclei are seen in bright blue. When CaCo2 cells were exposed to trophozoites (+Eh) or to ETEC (+ETEC, positive control), colocalization of the two fluorescent signals was revealed by a whitish color concentrated in the nuclei. Cells not exposed to pathogens (control cells) showed the p65 fluorescent green signal in the cytoplasm. Cells exposed to pathogens in presence of the inhibitors of the TLR/NFκB pathway (Eh+Bay, Eh+IMG, ETEC+Bay, ETEC+IMG) showed a distribution pattern of the p65 subunit similar to that of non- activated cells (control cells).

These results showed that trophozoites induced the expression of HBD2 mRNA via activation of TLRs on the surface of CaCo2 cells. Induction by ETEC, our positive control, occurred via the same signaling pathway. The activation of this pathway, by the presence of the pathogens, was further confirmed by the immunofluorescence results showing the translocation of the activated p65 NFκB subunit to the cell nuclei. This process is known to occur in intestinal cells exposed to pathogenic bacteria [30].

### *Entamoeba histolytica* trophozoites induce production and release of HBD2 by CaCo2 cells

**Figure 2:**
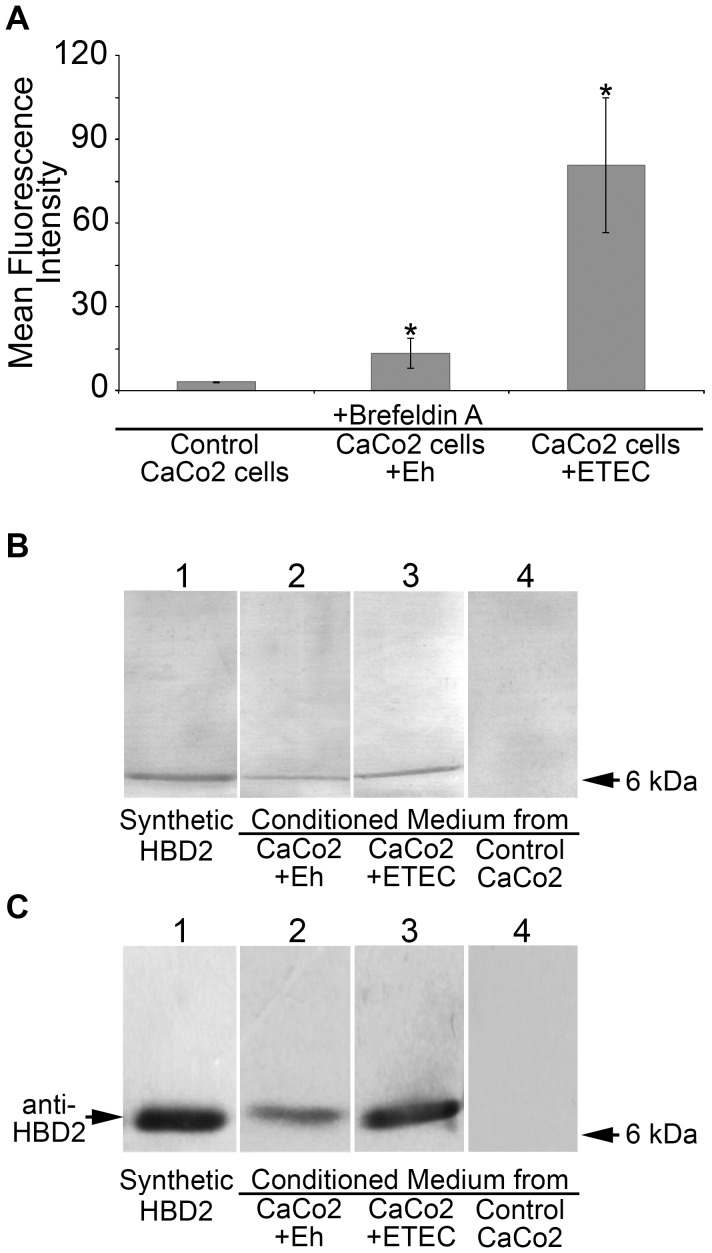
Production and release of HBD2 by CaCo2 cells exposed to *Entamoeba histolytica* trophozoites. A. Quantification of HBD2-positive CaCo2 cells after exposure to pathogens. CaCo2 cells were exposed for 2 h to ETEC or PFA-fixed trophozoites in the presence of 1.0 µg/ml Brefeldin A. Cells were permeabilized and fixed and then labeled with a specific anti-HBD2 antibody tagged with FITC. Levels of HBD2 inside the CaCo2 cells were determined by flow cytometry and data are presented as mean fluorescence intensity (MFI). Asterisks indicate statistical differences relative to control CaCo2 cells in three experiments done in triplicate (*P* value<0.01). B. Detection of HBD2 in cultured media from CaCo2 cells. Culture media were analyzed by SDS-PAGE in 15% gels. A silver-stained representative gel is shown. Lane 1, synthetic HBD2 peptide. Lane 2, CM from CaCo2 cells exposed to PFA-fixed *E. histolytica* trophozoites (CaCo2+Eh). Lane 3, CM of ETEC-exposed CaCo2 cells (CaCo2+ETEC). Lane 4, CM from CaCo2 cells not exposed to pathogens (Control CaCo2). C. Immunodetection of HBD2 in a parallel gel to the one shown in panel B. After electrophoresis, proteins were blotted onto nitrocellulose membranes and challenged with anti-HBD2 antibody. Lane 1, synthetic HDB2 peptide. Lane 2, CM of CaCo2 cells exposed to PFA-fixed *Entamoeba histolytica* trophozoites (CaCo2+Eh). Lane 3, CM of CaCo2 cells exposed to ETEC (CaCo2+ETEC). Lane 4. CM of CaCo2 cell cultures not exposed to pathogens (Control CaCo2).

The above results demonstrated that exposure of CaCo2 cells to PFA-fixed *E. histolytica* trophozoites induced a significant augment of HBD2 mRNA expression. To determine whether the corresponding peptide was present in CaCo2 cells exposed to PFA-fixed trophozoites or to ETEC, Brefeldin A (BFA) was added to the cultures at a final concentration of 1.0 µg/ml at the same time as cells were exposed to pathogens to inhibit protein transport and release into CM. Cells not exposed to pathogens were the negative control. After incubation for 2 h and removal of pathogens, CaCo2 cells were washed several times with medium without BFA and treated with the permeabilizing solution as indicated in Methods. Cells were then incubated with anti-HBD2 antibody and a FITC-tagged secondary antibody and analyzed by flow cytometry. Results in Figure 2A show the mean fluorescence intensity (MFI) measured as arbitrary units in 10,000 cells in each condition. HBD2-positive cells exposed to trophozoites showed an MFI value of 15, whereas cells exposed to ETEC showed a value of 70 and control cells (not exposed to pathogens) showed a value of 4.

To assess if HBD2 produced by CaCo2 cells exposed to pathogens was being released, CM were collected from these cells and from control cells. Conditioned media were concentrated by evaporation afterwards we added 10× Laemmli electrophoresis buffer and subjected to SDS-PAGE in 15% gels. Figure 2B shows a representative gel stained with silver nitrate. Lane 1 loaded with 100 ng of synthetic HBD2 revealed a single band with an electrophoretic mobility of approximately 5 kDa. Lanes 2 and 3 were loaded with 150 µl of CM from CaCo2 cells exposed to trophozoites or to ETEC, respectively, containing 60 µg of total protein. In these two lanes, a protein with the same electrophoretic mobility as the band observed for synthetic HBD2 was revealed. In lane 4, CM from cells not exposed to pathogens, also containing 60 µg of total protein, were loaded. In this lane, a band in the 5-kDa region was not observed. Figure 2C shows the immunoblot of a parallel gel challenged with the anti-HBD2 polyclonal antibody. A single band around 5 kDa in lanes 1, 2 and 3 was the only antibody-positive band, while none was detected in lane 4. Densitometry analysis of the 5 kDa protein band, detected in three representative silver-stained gels and representative Western blots, indicated that HBD2 in CM of ETEC-exposed CaCo2 cells corresponded to 90.37±13.17 ng and HBD2 in CM of trophozoite-exposed CaCo2 cells corresponded to 37.92±7.32 ng. These results showed that HBD2 induced in CaCo2 cells by pathogens is released into CM and that peptide levels released by trophozoite-exposed CaCo2 cells were approximately half of those released by cells exposed to ETEC.

### *Entamoeba histolytica* trophozoites are susceptible to permeabilization and cell damage by HBD2 secreted by pathogen-exposed CaCo2 cells

**Figure 3:**
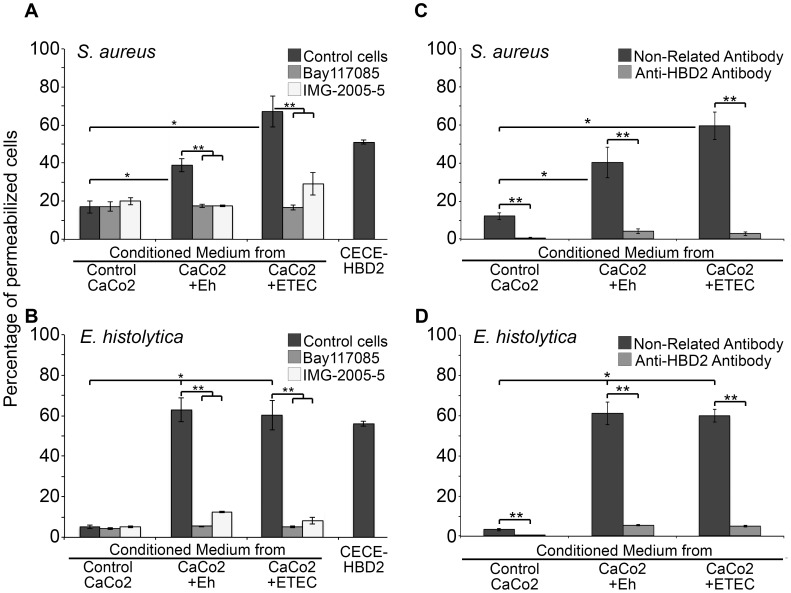
Permeabilizing effect of HBD2 released by CaCo2 cells after exposure to *Entamoeba histolytica* trophozoites. A. Permeabilization of *Staphylococcus aureus* bacteria, a known strain sensitive to HBD2 activity, by CM from CaCo2 cells exposed to pathogens. One hundred thousand bacteria from an overnight culture were exposed for 1 h to CM from CaCo2 cells exposed to ETEC (CaCo2+ETEC, positive control), to CM from cells exposed to PFA-fixed trophozoites (CaCo2+Eh) or incubated with 10 ng/ml of CECE-HBD2. Propidium iodide internalization into permeabilized bacteria was quantified by flow cytometry. B. Permeabilization of *E. histolytica* trophozoites by CM from CaCo2 cells exposed to pathogens. One hundred thousand trophozoites were exposed to CM from CaCo2 cells exposed to ETEC (CaCo2+ETEC, positive control), to CM from cells exposed to PFA-fixed trophozoites (CaCo2+Eh) or incubated with 10 ng/ml of CECE-HBD2. In parallel, CM from CaCo2 cell cultures also exposed to either pathogen and incubated with inhibitors of the TLR2/4-NFκB pathway, Bay117085 and IMG-2005-5, were used to permeabilize *S. aureus* bacteria or *E. histolytica* trophozoites. Propidium iodide internalization into trophozoites was quantified as indicated for bacteria. C and D. Neutralization of HBD2 activity present in CM. Media obtained from CaCo2 cell cultures exposed to pathogens were neutralized with anti-HBD2 antibody (2.0 µg/ml for 2 h); then, *S. aureus* bacteria or *E. histolytica* trophozoites were incubated with these neutralized media. As control, a non-related polyclonal antibody (anti-human Sirt1) was used. Permeabilization levels were evidenced by propidium iodide penetration and quantified by flow cytometry. Data for all panels are presented as percentage of permeabilized cells ± SD. * indicates differences between control cells and cells exposed to pathogens. ** Indicates statistical differences between inhibited (panels A and B) or neutralized (panels C and D) conditions versus values obtained in non-inhibited or non-neutralized conditions in three experiments done in triplicate (*P* value<0.01).

Our results above showed for the first time that CaCo2 cells exposed to *Entamoeba histolytica* trophozoites induced the expression of HBD2 mRNA and a peptide with similar molecular features to HBD2 produced by CaCo2 cells, as previously reported in an ETEC-induction model [30]. Therefore, a functional assay was carried out to demonstrate that trophozoite-induced HBD2 has the capability to permeabilize bacteria and amebas. For this, propidium iodide penetration into pathogens was used as the indicator of permeabilization. This fluorescent dye enters to membrane-damaged cells and binds to double strand DNA, but it is excluded from cells with intact membranes. *Staphylococcus aureus* bacteria, known to be sensitive to HBD2 [24], [26], were utilized to test the antimicrobial activity of CM obtained from pathogen-exposed Caco2 cells. As synthetic HBD2 could not be used in this assay, because the antimicrobial activity requires of three functional S-S bonds that are present in the native molecule, we prepared an enriched fraction of the ETEC-induced HBD2 (CECE-HBD2) to be used as the positive control for cell permeabilization activity. Figure 3A shows the results obtained by flow cytometry quantification of propidium iodide inside bacteria incubated in CM in the different conditions. Conditioned media from cells exposed to trophozoites caused permeabilization of 38% of bacteria. Conditioned media from ETEC-exposed CaCo2 cells caused permeabilization of 63.8% of bacteria. CECE-HBD2 (10 ng/ml) caused permeabilization of 54% of them, while bacteria incubated with CM from control cells (not exposed to pathogens) internalized propidium iodide at low levels (18%). A similar value was determined for bacteria incubated in CM from cells exposed to pathogens in the presence of inhibitors of the classic TLR pathway. These results demonstrated that HBD2 induced by trophozoites has the capacity to permeabilize sensitive bacteria. The higher levels of permeabilization caused by CM obtained from ETEC-exposed CaCo2 cells seem related to the higher HBD2 levels found in these media.

An assay under the same conditions was performed to test the antimicrobial activity of CM and CECE-HBD2 (as positive control) on live trophozoites. Figure 3B shows that 50% to 60% of the trophozoites incubated with CECE-HBD2 or with CM from CaCo2 cells exposed to pathogens were permeabilized. As in the case of bacteria, the percentage of permeabilized trophozoites incubated in CM from control CaCo2 cells or in CM from cells treated with the inhibitors of TLR/NFκB pathway, only reached values in the range of 5% to 12%. These results showed that CM from CaCo2 cells exposed to trophozoites could permeabilize live trophozoites in percentages similar to those observed using CM from ETEC-exposed cells. The unexpected result for the high percentage of trophozoites being permeabilized by a lower concentration of HBD2 in CM from trophozoite-exposed CaCo2 cells could be explained by a higher liability of trophozoite membranes or by the possible presence in these CM of other molecules that contribute to trophozoites damage.

To test the participation of HBD2 in the membrane damage inflicted to trophozoites, similar permeabilization assays were performed as indicated above, but instead of inhibitors, 2 µg/ml of a polyclonal anti-HBD2 antibody were added to the CM. Figures 3C and 3D show that CM from cells exposed to pathogens, that had been preincubated with the anti-HBD2 antibody, lost their capacity to permeabilize either bacteria or live trophozoites. In contrast, CM containing an irrelevant polyclonal antibody (anti-human Sirt1) did not significantly reduce their capacity to permeabilize pathogens. These results provided support for HBD2 being the main component in the CM responsible for the damage inflicted on bacteria and trophozoites, although other molecules, not yet identified present in CM or released by permeabilized amebas, could contribute to increase the initial damage caused by HBD2.

**Figure 4:**
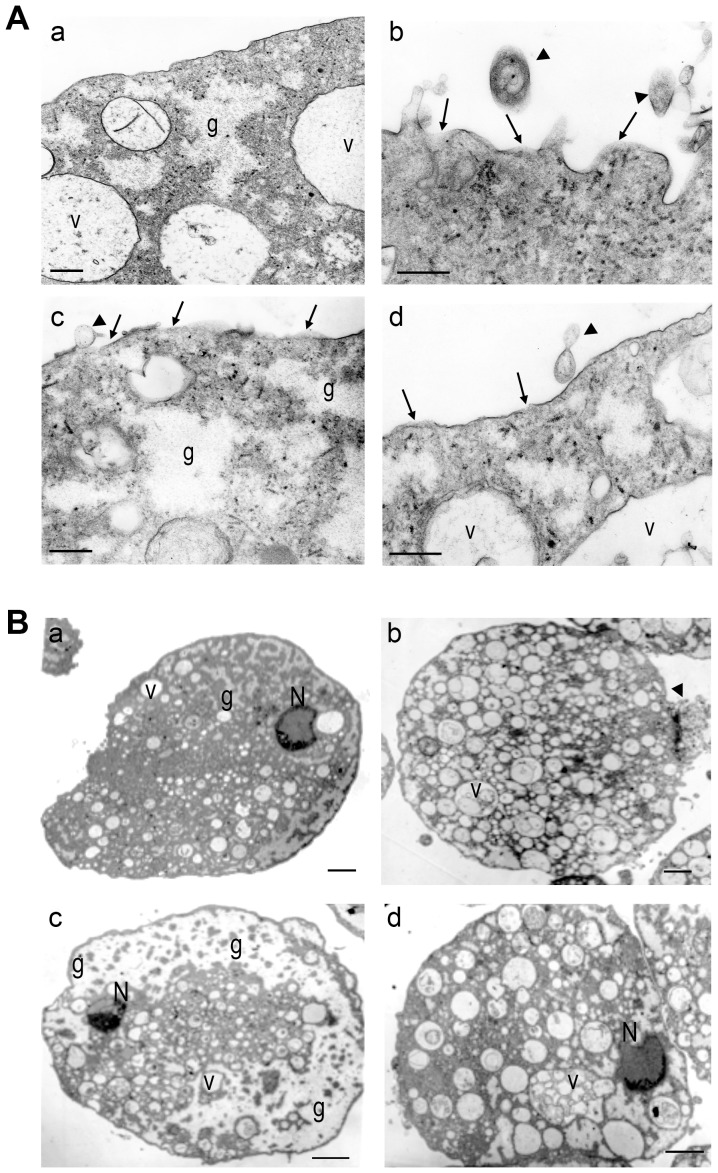
Ultrastructural alterations of trophozoites assessed by transmission electron microscopy. In panels A and B, *E. histolytica* trophozoites were incubated in: CM of CaCo2 cells not exposed to the pathogens (a); CM from cells exposed to PFA-fixed trophozoites (b); CM from cells exposed to ETEC (c) or incubated with 10 ng/ml of CECE-HBD2 (d) and then prepared for transmission electron microscopy. Panel Aa, a control trophozoite showing continuous plasma membrane without alterations (arrows), small clear zones in the cytoplasm in which glycogen localizes (g) and clear vacuoles (v). Panel Ab shows a trophozoite incubated in CM from CaCo2 cells exposed to trophozoites with many alterations in the cytoplasm, ruptured zones in the plasma membrane and extruded cellular material and a great increase in the number of vacuoles containing cellular material (v). Panel Ac shows a representative trophozoite exposed to CM from ETEC-exposed cells with discontinuities in the membrane and ruptures (arrows), increased content of glycogen (g) and increased number of vacuoles, many containing cellular material (v). Arrowheads point to extracellular material. Panel Ad, a trophozoite treated with CECE-HBD2 shows alterations of the membranes similar to those observed in trophozoites incubated in CM from pathogen-exposed cells. Bar = 0.5 µm. B. Low magnification of representative trophozoites treated as indicated in each of the panels above, shows control trophozoites with regular distribution of the chromatin (N), tethered to the nuclear membrane. Trophozoites treated with CM of pathogen-exposed cells or CECE-HBD2 show chromatin aggregated and polarized (N) and a high number of big vacuoles containing cellular material (v). Bar = 3.0 µm.

HBD2 has been reported to damage other protozoan parasites initiating cell destruction by making perforations in their membrane [23]. Considering this antecedent and the above results, we analyzed the ultrastructural integrity of trophozoites incubated in CM from pathogen-exposed CaCo2 cells, from control trophozoites and trophozoites incubated with CECE-HBD2. Figure 4A (panel a) depicts a representative trophozoite incubated in CM from control CaCo2 cells. It shows a continuous plasma membrane, the typical small accumulations of cytoplasmic glycogen (g) and well-formed vacuoles with clear content and continuous membranes (v). In contrast, trophozoites incubated in CM from CaCo2 cells exposed to *Entamoeba histolytica* (panel b) or to ETEC (panel c), as well as trophozoites incubated with 10 ng/ml of CECE-HBD2 (panel d) showed membranes, both plasmatic and vacuolar with multiple discontinuous zones and irregular width that could correspond to pores (arrows); together with several ruptured points and release of cellular material can be seen (arrowheads). Also evident, is the increased accumulation of glycogen in the cytoplasm (g). Figure 4B shows low magnification micrographs of trophozoites treated as indicated in figure 4A. In panels b, c and d, it is possible to appreciate a notorious increase in the number of vacuoles (v) and glycogen accumulation (g), plus chromatin aggregation and its polarization inside the nucleus (N), and other signs of cell lysis, such as the extruded cytoplasm in one side of the trophozoite (Figure 4Bb, arrowhead). These alterations were not observed in trophozoites incubated in CM from CaCo2 cells not exposed to pathogens (panel a) nor in those incubated in CM from cells exposed to TLR classical pathway inhibitors (not shown). These results corroborated that trophozoite-induced HBD2 in CaCo2 cells has the capacity to permeabilize bacteria and trophozoite membranes. Membrane damage in trophozoites leads to loss of cell integrity and their destruction.

## Discussion

Diverse mechanisms of innate immunity response against pathogens have been described in the literature. One of them is the production of antimicrobial peptides, a defense process that is present from primitive organisms up to mammals [20], [21], [22]. The intestinal mucosa is one of the tissues that are constantly challenged by the presence of pathogens. Its immune innate response is an important barrier to avoid infection and damage of the organism by microbes. Bacterial ligand-mediated induction of TLR signaling in colonocytes, via activation of the transcription factor NFκB, induces expression and secretion not only of pro-inflammatory cytokines [9], but also of antimicrobial peptides such as HBD2 and HBD3 into the extracellular milieu [29], [33]. Molecules such as bacterial LPS from Gram-negative bacteria that binds to TLR-4, peptidoglycan (PGN) from Gram-positive bacteria that binds to TLR-2, enterotoxins and even commensal bacteria components can induce secretion of defensins, particularly of HBD2 [33], [34], [35].

Recent studies with *E. histolytica* have shown that trophozoites bind to TLR-2 and TLR-4 in human colonic cells through the carbohydrate recognition domain of the Gal/GalNac lectin and the lipopeptidophophoglycan present in the parasite surface. By acting as a bacterial PAMP, these amebic molecules activate the classical TLR signaling pathway inducing NFκB activation, increased expression of TLRs and increased secretion of inflammatory cytokines [9], [36]. Induction of an intestinal inflammatory response by trophozoites has been proposed as an important factor to determine the course of the parasite infection [37]. Nevertheless, little is yet known about the innate immune response induced by trophozoites in the human intestine and how this could affect the intestinal microbiota, particularly when other intestinal pathogens are present To our knowledge, this is the first report showing that amebic interaction with human intestinal cells leads to production of a defensin peptide that have antimicrobial activity against pathogenic bacteria and to amebas. Hence, further knowledge about the molecular and immunologic mechanisms induced by trophozoites and how these are modulated by their interaction with the host intestinal microbiota, are important aspects to understand the course of infections and tissue invasion.

We showed in this work, that exposure of CaCo2 cells to *E. histolytica* trophozoites induced expression and release of a peptide of approximately 5 kDa with identical electrophoretic mobility and immunoreactivity to synthetic HBD2 and to that obtained from CaCo2 cells exposed to ETEC [30], which we utilized as positive control in all the assays of this work. Our experimental approach demonstrated that trophozoite- or ETEC-induced HBD2 mRNA expression was sensitive to inhibitors of Myd88 and NFκB, two of the main effectors in the classic TLR pathway in proinflammatory cytokine induction. The lower expression of HBD2 mRNA induced by trophozoites compared to the higher induction by ETEC may be explained by: 1) lower affinity of amebic ligands (specifically the Gal/GalNac lectin) to CaCo2 cell TLRs, derived from the necessary PFA-fixation of trophozoites used for the induction of HBD2; or 2) an induction initiated by molecules other than TLR ligands present in ETEC with capacity to activate NFκB, as has been reported for the flagellar protein FliC of *Salmonella* [38]. However, lack of induction of HBD2 mRNA by CaCo2 cells in the presence of inhibitors of NFκB and MyD88 activity, provides support for an induction via TLRs. Furthermore, our previous work, using PFA-fixed trophozoites, showed that these could bind to the cells and activate TLR signaling pathways in a similar way as was done by several pathogenic and non-pathogenic bacteria [9]. As a possible consequence of the lower induction of HBD2 mRNA in CaCo2 cells exposed to trophozoites, a lower production of the peptide could be expected, when a comparison was made with HBD2 levels induced in cells exposed to ETEC. Although the antimicrobial activity of defensins is mainly directed to destroy bacteria by permeabilization of their membrane, other pathogens, among which are protozoan parasites, are labile to human defensins [23], [24]. The activity of the trophozoite-induced antimicrobial peptide, tested by permeabilization of HBD2-sensitive *Staphylococcus aureus* and neutralization assays, corroborated its identity as a functional HBD2. Moreover, despite the lower levels of the defensin induced by trophozoites, they were sufficient to inflict permeabilization of a significant percentage of bacteria and damage of a similar percentage of live trophozoites. Conversely, damage was not produced to trophozoites incubated in CM from control cells or in conditions where the expression or activity of HBD2 were blocked by inhibitors or by the specific anti-HBD2 antibody.

The higher liability of live trophozoites to HBD2 present in CM, compared with the liability of bacteria could result from the molecular composition of trophozoite membranes and in particular from their specific lipid composition that allows continuous translocation of surface molecules [39]. The permeabilization assays and the electron microscopy analysis of trophozoites exposed to pathogen-CM showed that the parasite membranes were rapidly damaged and followed by cell destruction. Furthermore, the results showed that trophozoite damage was mostly dependent on HBD2 activity, since it was significantly reduced in the presence of an HBD2-neutralizing antibody.

A recent report has shown that *E. histolytica* trophozoites induce intestinal cathelicidin mRNA and the protein expression in HT-29 cells and that trophozoites were refractive to damage by this antimicrobial peptide, while bacteria were not [40]. Therefore, cathelicidins could not be contributing to the damage of trophozoites that we have observed in the present work. All these together contribute to the proposal that considers pathogen interplay as an important factor in intestinal infections. In a very simplistic way, we could hypothesize that: if trophozoites can induce synthesis of both types of antimicrobial peptides and are resistant to cathelicidins, but sensitive to β defensins, while bacteria are sensitive to both, these peptides could act in concert to select particular pathogens. On one side, killing bacteria by cathelicidins would facilitate amebic colonization and invasion whereas; killing amebas by defensins would create an opportunity for resistant bacteria to proliferate. Furthermore, destruction of trophozoites by defensins would increase release of amebic cysteine proteinases. These, besides breaking down cathelicidins, can activate pro-inflammatory cytokines released by the intestinal cells and synergize pathogen-induced inflammation [9], [39], [41]. Furthermore, it has been reported that not only pathogenic bacteria induce expression of defensins in CaCo2 cells, but that this is also done by a variety of probiotic bacteria, including non-pathogenic strains of *Escherichia coli and* lactobacilli [15], [35]. In the human intestine, several types of bacteria are regularly present either as commensals or as pathogens, all of them potential inductors of defensin expression together with an inflammatory response. In the above hypothetical conditions, although many pathogens will be eliminated, the inflammatory milieu generated could be exacerbated, as defensins and amebic and bacterial components also contribute to inflammation by recruiting immune cells [42], [43], [44]. In addition, disruption of the barrier function of the intestinal epithelium by pathogen-induced inflammation will allow penetration of normally excluded molecules and more pathogens into the inflammation sites [8], [10], [45]. The course of an intestinal infection in these conditions would favor pathogen invasion.

Recent studies of intestinal pathogens and the intestinal microbiota of the host have shown the importance of their relationship in inflammatory and infectious diseases of the human intestine (reviews in The Gut Microbiota issue, Science, 336, 2012). In spite of the complexity of in vitro intestinal cell models to study pathogen interplay they still provide a unique opportunity to identify factors and mechanisms underlying the severity and spreading of diseases such as Amebiasis.

## Supporting Information

Figure S1 **Description and characterization of oligonucleotide primers used for the quantitative determination of HBD2 mRNA expression.** A. Sequences of HBD2 (*defb4a*) and *rplp0* genes. In bold are indicated the annealing sites for the primers used in the quantitative relative RT-PCR and underlined letters indicate the expected PCR product for each pair of primers. B. Dissociation curves of PCR products for each pair of primers for *defb4a* and *rplp0* showing only one main PCR product. Data are presented as derivative of the fluorescence intensity relative to the temperature. C. Electrophoretic separation of PCR products for *defb4a* and *rplp0* genes. After PCR, products were separated in a 1.5% agarose gel, stained with ethidium bromide and documented (bp: base pair). D. Comparison of Ct values of the endogenous control gene *rplp0* under the different experimental conditions reported in this work. Control: CaCo2 cells alone. +ETEC: CaCo2 cells exposed to ETEC. +Eh: CaCo2 cells exposed to PFA-fixed *E. histolytica* trophozoites. +IMG: CaCo2 cells incubated with IMG-2005-5. +Bay: CaCo2 cells incubated with Bay117085. Data were analyzed by 2-way ANOVA (*P* = 0.84).
(TIF)
