# Supplementary figures and images for: Toll-like Receptor Signaling Activation by Entamoeba histolytica Induces Beta Defensin 2 in Human Colonic Epithelial Cells: Its Possible Role as an Element of the Innate Immune Response
Source: PLoS Negl Trop Dis. 2013 Feb 28;7(2):e2083. doi: 10.1371/journal.pntd.0002083 (PMC3585038; doi:10.1371/journal.pntd.0002083)

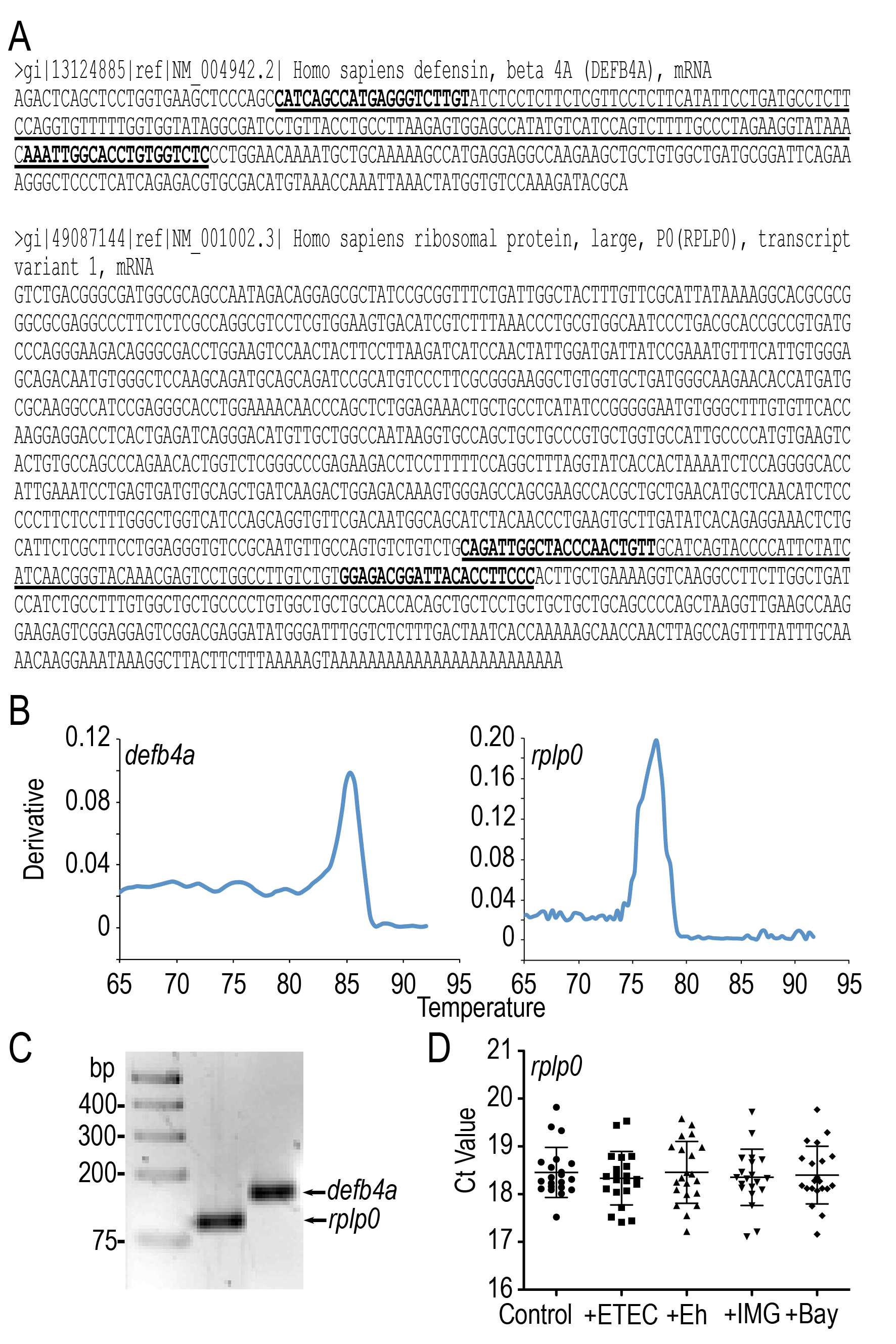

Supplement: Figure S1 — Description and characterization of oligonucleotide primers used for the quantitative determination of HBD2 mRNA expression. A. Sequences of HBD2 (defb4a) and rplp0 genes. In bold are indicated the annealing sites for the primers used in the quantitative relative RT-PCR and underlined letters indicate the expected PCR product for each pair of primers. B. Dissociation curves of PCR products for each pair of primers for defb4a and rplp0 showing only one main PCR product. Data are presented as derivative of the fluorescence intensity relative to the temperature. C. Electrophoretic separation of PCR products for defb4a and rplp0 genes. After PCR, products were separated in a 1.5% agarose gel, stained with ethidium bromide and documented (bp: base pair). D. Comparison of Ct values of the endogenous control gene rplp0 under the different experimental conditions reported in this work. Control: CaCo2 cells alone. +ETEC: CaCo2 cells exposed to ETEC. +Eh: CaCo2 cells exposed to PFA-fixed E. histolytica trophozoites. +IMG: CaCo2 cells incubated with IMG-2005-5. +Bay: CaCo2 cells incubated with Bay117085. Data were analyzed by 2-way ANOVA (P = 0.84). (TIF) [file pntd.0002083.s001.tif]
